# Nickel Decorated on Phosphorous-Doped Carbon Nitride as an Efficient Photocatalyst for Reduction of Nitrobenzenes

**DOI:** 10.3390/nano6040059

**Published:** 2016-04-01

**Authors:** Anurag Kumar, Pawan Kumar, Chetan Joshi, Manvi Manchanda, Rabah Boukherroub, Suman L. Jain

**Affiliations:** 1 CSIR Indian Institute of Petroleum, Haridwar road Mohkampur, Dehradun 248005, India; anuragmnbd@gmail.com (A.K.); choudhary.2486pawan@yahoo.in (P.K.); chetanjoshi019@gmail.com (C.J.); manvimanchanda@gmail.com (M.M.); 2 Academy of Scientific and Industrial Research (AcSIR), New Delhi 110001, India; 3 Institut d’Electronique, de Microélectronique et de Nanotechnologie (IEMN), UMR CNRS 8520, Université Lille1, Avenue Poincaré-BP 60069, 59652 Villeneuve d’Ascq, France

**Keywords:** carbon nitride, nickel nanoparticles, photocatalysis, visible light, nitrobenzene reduction

## Abstract

Nickel nanoparticle-decorated phosphorous-doped graphitic carbon nitride (Ni@g-PC_3_N_4_) was synthesized and used as an efficient photoactive catalyst for the reduction of various nitrobenzenes under visible light irradiation. Hydrazine monohydrate was used as the source of protons and electrons for the intended reaction. The developed photocatalyst was found to be highly active and afforded excellent product yields under mild experimental conditions. In addition, the photocatalyst could easily be recovered and reused for several runs without any detectable leaching during the reaction.

## 1. Introduction

Solar energy is abundant, inexpensive and has great potential for use as a clean and economical energy source for organic transformations [1,2,3,4]. Thus, the efficient conversion of solar energy to chemical energy, *i.e.*, visible light-driven chemical conversion (photocatalysis) is an area of tremendous importance and has been rapidly growing worldwide during the last two decades. The reduction of nitrobenzenes to the corresponding amines is an important transformation both in organic synthesis as well as in the chemical industry [5,6,7]. The conventional methods for this reaction mainly require harsh reaction conditions, expensive reagents, multi-step synthetic procedures and mostly generate hazardous waste [8,9]. Owing to the current need to develop greener and sustainable synthesis, the visible light-assisted reduction of nitrobenzenes to the corresponding amines is highly desired, as these reactions occur under mild and ambient conditions [10]. So far, semiconducting materials, mainly TiO_2_-based heterogeneous photocatalysts, have been widely used for the reduction of nitrobenzenes [11]. However, these photocatalysts work only under ultraviolet (UV) irradiation, which is a small part of the solar spectrum and also needs special reaction vessels. In order to improve their efficiency in the visible region, surface modification of the TiO_2_ photocatalyst by doping metal or metal oxides, oxide halides *(i.e.*, PbPnO_2_X (Pn = Bi, Sb; X = Br, Cl) and sensitization with dyes has also been demonstrated [12,13,14]. However, the tedious synthetic procedures and poor product yields limit the synthetic utility of these methods.

Another semiconducting material, *i.e.*, carbon nitride, has gained significant attention in recent years due to its low band gap (2.7 eV) and easy synthesis method [15,16,17]. The graphitic carbon nitride (g-C_3_N_4_) is a two dimensional (2D) polymer consisting of interconnected tri-s-triazine units via tertiary amines. The low band gap and the appropriate position of the conduction (−1.1 eV) and valence (+1.6 eV) bands enable it to initiate any redox reaction [18]. Despite of several advantages, it also suffers from certain drawbacks such as only being able to absorb blue light up to the 450 nm wavelength, so the red region of visible light cannot be harvested. In order to widen the absorption profile in the whole visible region, the doping of elements such as phosphorus (P), boron (B), fluorine (F), etc is important because these atoms form bonds with nitrogen and contribute their electrons to the π-conjugated system more efficiently. Recently, Zhang *et al.* synthesized P-doped g-C_3_N_4_ with an improved photocurrent response by using 1-butyl-3-methylimidazolium hexafluorophosphate (BmimPF_6_) as a source of phosphorous [19]. Hong and coworkers synthesized sulfur-doped mesoporous carbon nitride (mpgCNS) using an *in situ* doping method, which showed increased photocatalytic performance for the solar hydrogen production [20]. Wang *et al.* synthesized fluorine-doped carbon nitride by using ammonium fluoride as a cheap source of fluorine for the enhanced visible light-driven hydrogen evolution reaction [21]. Very recently, our group reported an iron(II) bipyridine complex grafted nanoporous carbon nitride (Fe(bpy)_3_/npg-C_3_N_4_) photocatalyst for the visible light-mediated oxidative coupling of benzylamines to imines [22].

**Scheme 1:**
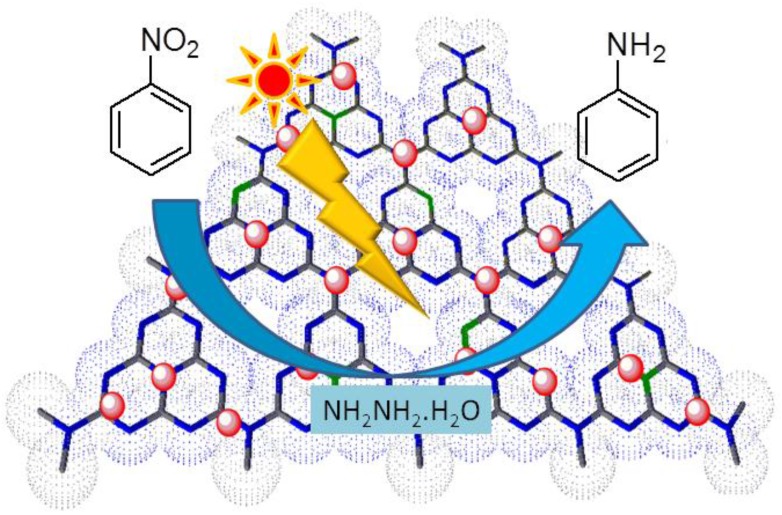
Reduction of nitrobenzenes on Ni@g-PC_3_N_4_ (nickel nanoparticles grafted on P-doped g-C_3_N_4_) catalyst.

In continuation of our ongoing research on the development of novel photocatalysts [23,24,25], herein we report for the first time on nickel nanoparticles grafted on P-doped g-C_3_N_4_ (Ni@g-PC_3_N_4_) as a high-performance photocatalyst for the reduction of nitrobenzenes to the corresponding amines at ambient temperature under visible light irradiation (Scheme 1). Due to better visible light absorption, P-doped carbon nitride can generate electron-hole pairs under visible light irradiation, while nickel nanoparticles, due to their lower Fermi level than conduction band of P-C_3_N_4_, can work as electron capturing agents to slow down the process of electron-hole recombination and enhance the catalytic activity and reaction rate.

## 2. Results and Discussion

### 2.1. Synthesis and Characterization of the Photocatalyst

**Scheme 2:**
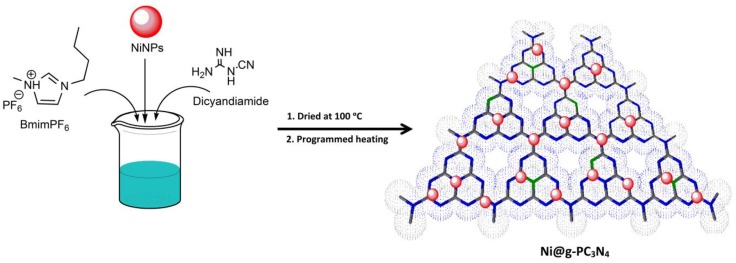
Synthetic illustration of Ni@g-PC_3_N_4_ catalyst. NiNPs: nickel nanoparticles; BmimPF_6_: 1-butyl-3-methylimidazolium hexafluorophosphate.

At first, nickel nanoparticles were synthesized by reducing nickel chloride hexahydrate by sodium borohydride in the presence of cetyltrimethylammonium bromide (CTAB) as a template by following the literature procedure in [26]. Next, the synthesis of phosphorous-doped graphitic carbon nitride (g-PC_3_N_4_) was performed by using ionic liquid 1-butyl-3-methylimidazolium hexafluorophosphate [BmimPF_6_] as a source of phosphorous and dicyandiamide as a precursor for the graphitic carbon nitride skeleton [17,27]. Nickel nanoparticle-decorated phosphorous-doped carbon nitride (Ni@g-PC_3_N_4_) with variable nickel content (2–7.5 wt %) was synthesized by the addition of nickel nanoparticles, BmimPF_6_ and dicyandiamide into water with continuous stirring. The resulting suspension was heated at 100 °C until all the water was evaporated. Then, the obtained solid was heated at a programmed temperature in a similar manner to the one used for P-doped carbon nitride (Scheme 2). Bare graphitic carbon nitride (g-C_3_N_4_) was synthesized for comparison by heating dicyandiamide under identical programmed temperature. Among the various nanocomposites synthesized, 5% nickel nanoparticles grafted on P-doped g-C_3_N_4_ (5%Ni@PC_3_N_4_) exhibited the best performance for the photocatalytic reduction of nitrobenzene; thus, we have used this photocatalyst for detailed characterization and further studies.

**Figure 1:**
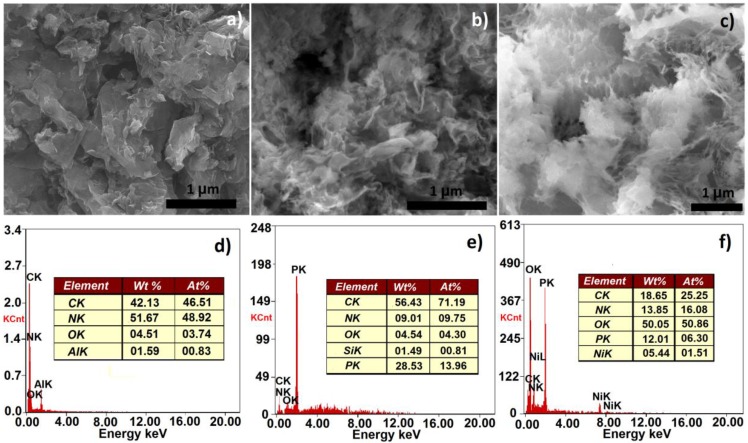
Field emission scanning electron microscopy (FE-SEM) image of: (**a**) graphitic carbon nitride (g-C_3_N_4_); (**b**) phosphorous-doped graphitic carbon nitride (g-PC_3_N_4_); (**c**) nickel nanoparticle-grafted g-PC_3_N_4_ (Ni@g-PC_3_N_4_); energy dispersive X-ray spectroscopy (EDX) pattern of: (**d**) g-C_3_N_4_; (**e**) g-PC_3_N_4_; (**f**) 5%Ni@g-PC_3_N_4_.

The surface morphology of the synthesized materials was determined by field emission scanning electron microscopy (FE-SEM). The graphitic carbon nitride (g-C_3_N_4_) showed many enfolded and crumpled sheet-like structures (Figure 1a). The framework of C_3_N_4_ contains nitrogen as a substituted heteroatom forming similarly to the π-conjugated system, as in graphitic planes, due to sp^2^ hybridization between carbon and nitrogen atoms. The phosphorous-doped carbon nitride (g-PC_3_N_4_) exhibits similar morphological characteristics (Figure 1b). In the case of nickel nanoparticle-grafted g-PC_3_N_4_ (5%Ni@g-PC_3_N_4_), a similar sheet-like morphology was observed that may be due to the incorporation of nickel nanoparticles between the graphitic carbon nitride sheets (Figure 1c). The energy dispersive X-ray spectroscopy (EDX) pattern of g-PC_3_N_4_ clearly showed the presence of phosphorous (Figure 1e) and, in 5%Ni@g-PC_3_N_4_, the peak due to nickel can clearly be seen, which confirmed the presence of both components in the synthesized photocatalyst (Figure 1f).

**Figure 2:**
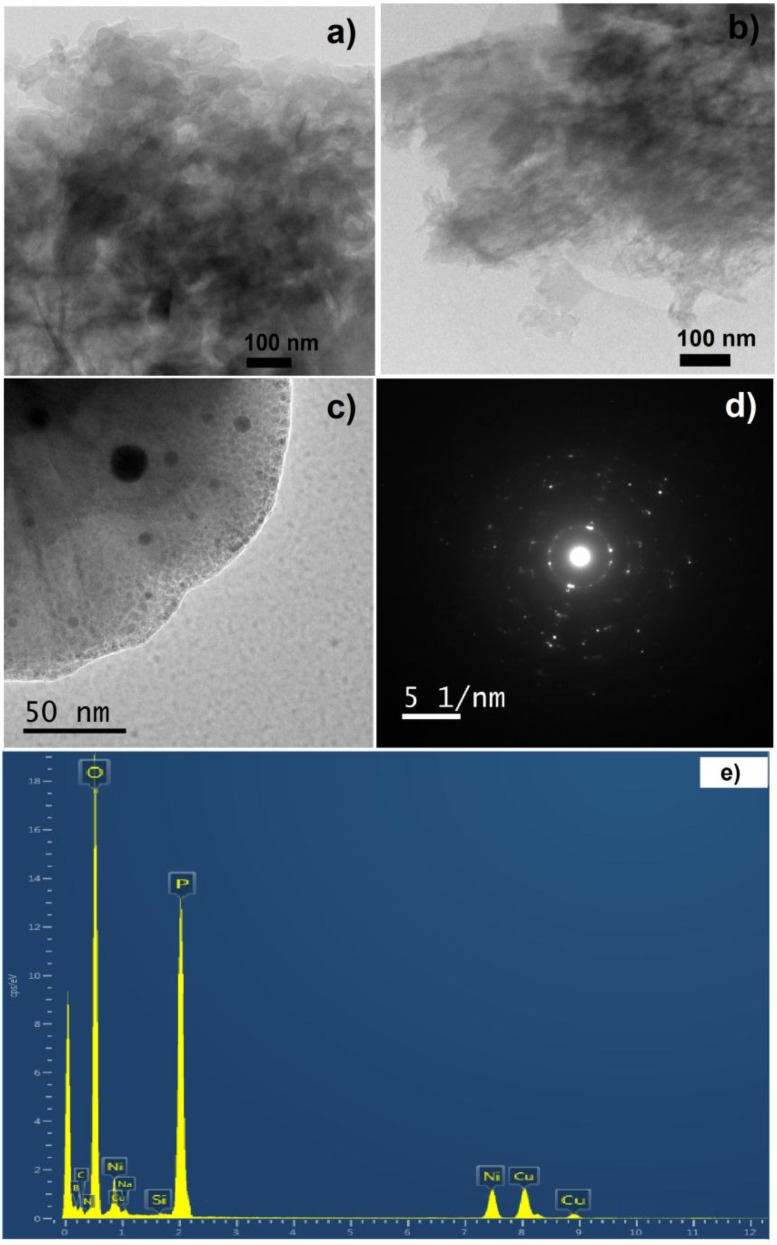
Transmission electron microscopy (TEM) images of: (**a**) g-C_3_N_4_; (**b**) g-PC_3_N_4_; (**c**) Ni@g-PC_3_N_4_; (**d**) selected area electron diffraction (SAED) pattern of Ni@g-PC_3_N_4_; (**e**) EDX pattern of Ni@g-PC_3_N_4_.

The fine morphological features of the synthesized materials were determined with the help of high-resolution transmission electron microscopy (HRTEM). The transmission electron microscopy (TEM) image of g-C_3_N_4_ at 100 nm resolution displayed enfolded sheets of carbon nitride (Figure 2a). For g-PC_3_N_4_, similar folded sheet-like structures were observed, indicating that phosphorus doping did not change the structural features of the material (Figure 2b). In the TEM image of 5%Ni@g-PC_3_N_4_, several small dark spots were observed, suggesting the presence of nickel nanoparticles between the scaffolds of graphitic structure of phosphorous-doped carbon nitride (Figure 2c). The presence of several rings and bright spots in the selected area electron diffraction (SAED) pattern of 5%Ni@g-PC_3_N_4_ confirmed the presence of nickel nanoparticles in the composite structure (Figure 2d). Further EDX patterns of 5%Ni@g-PC_3_N_4_ revealed the presence of all the expected elements in the photocatalyst (Figure 2e).

**Figure 3:**
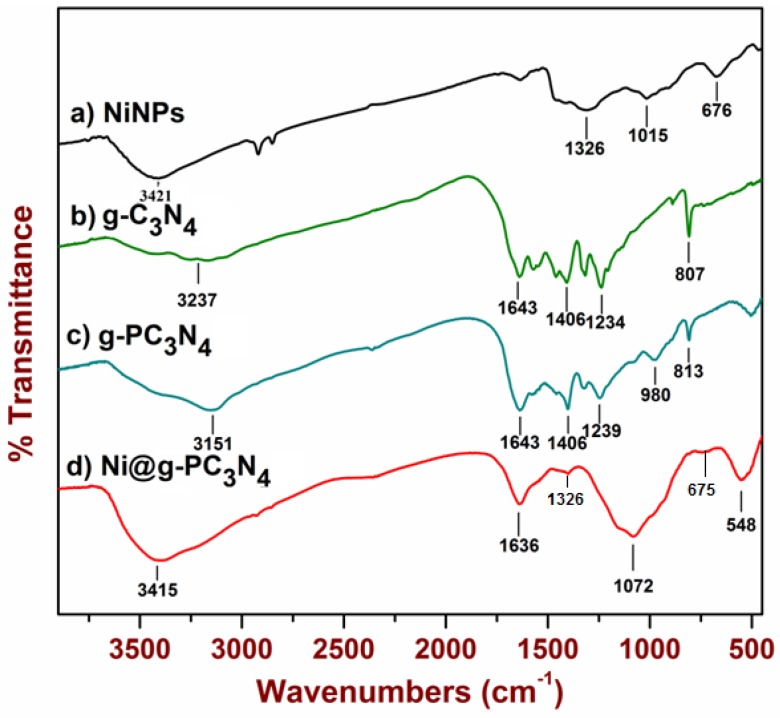
Fourier transform infrared (FTIR) spectra of: (**a**) nickel nanoparticles (NiNPs); (**b**) g-C_3_N_4_; (**c**) g-PC_3_N_4_; (**d**) 5%Ni@g-PC_3_N_4_.

The vibrational spectra of the synthesized materials are depicted in Figure 3. The Fourier transform infrared (FTIR) spectrum of as-synthesized nickel nanoparticles by using cetyl trimethylammonium chloride as a template was found to be well in agreement with the known literature and gave peaks at 676 cm^−1^ due to the Ni–O stretch of oxidized nickel [28]. Some additional peaks were also identified that may be due to the residual template molecules in the particles. The FTIR spectrum of carbon nitride (g-C_3_N_4_) showed a characteristic peak at 815 cm^−1^ related to C–N heterocycles due to the triazine ring mode. Another peak in the range of 1200–1600 cm^−1^, attributed to the specific aromatic skeleton vibration of carbon nitride, was observed [29]. A broad band was evident in the range of 3000–3700 cm^−1^ corresponding to adsorbed moisture, *i.e.*, the presence of water molecules, or due to the stretching mode of –NH_2_, which are uncondensed amine groups, or to N–H group vibrations present at the surface of carbon nitride (Figure 3a). For the P-doped carbon nitride (g-PC_3_N_4_), peaks related to C–N at 1520, 1440 and 1310 cm^−1^ were found to be diminished, which is mainly due to the displacement of some carbon atoms in the ring skeleton by the phosphorous atoms to give P–N bonds. Furthermore, a new peak at 980 cm^−1^ due to the vibration of the C–P heterocycle was clearly observed (Figure 3c). For the 5%Ni@g-PC_3_N_4_, the appearance of some peaks due to nickel nanoparticles with g-PC_3_N_4_ confirmed the successful synthesis of nickel-grafted P-doped g-C_3_N_4_ (Figure 3d).

**Figure 4:**
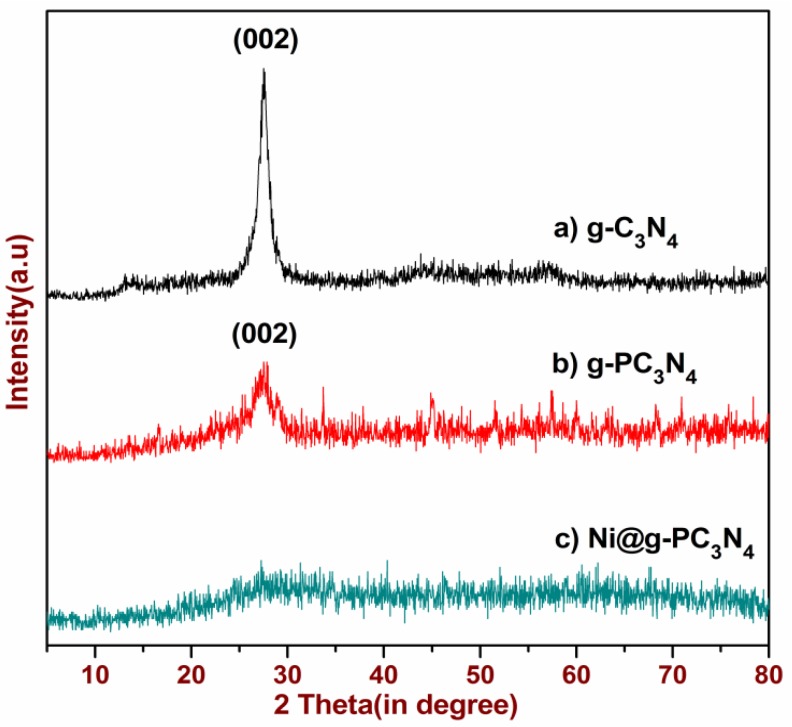
X-ray diffraction (XRD) patterns of: (**a**) g-C_3_N_4_; (**b**) g-PC_3_N_4_; (**c**) 5%Ni@g-PC_3_N_4_. a.u.: arbitrary units.

The crystallinity and phase structure of the nanocomposites were determined by X-ray diffraction (XRD) (Figure 4). The XRD diffraction pattern of graphitic carbon nitride (g-C_3_N_4_) revealed an intense broad peak at the 2θ value of 27.4°, indexed to (002) planes with 0.32 nm interlayer distance due to the stacking of graphite-like conjugated triazine aromatic sheets, which matches well with Joint committee on powder diffraction standards (JCPDS) 87–1526 for g-C_3_N_4_ (Figure 4a) [30]. The g-PC_3_N_4_ also exhibited an identical diffraction pattern; however, the intensity of the peak at 27.4° was found to be reduced due to the replacement of carbon atoms by phosphorous atoms (Figure 4b). The diffraction pattern of the 5%Ni@g-PC_3_N_4_ did not show any peak of nickel nanoparticles. This is most likely due to the amorphous nature of the material (Figure 4c). Furthermore, the peak at 27.4° was absent in 5%Ni@g-PC_3_N_4_, which was most probably due to the intercalation of nickel nanoparticles between the sheets. Therefore, the interlayer distance between sheets increased and the diffraction due to stacked sheets disappeared.

**Figure 5:**
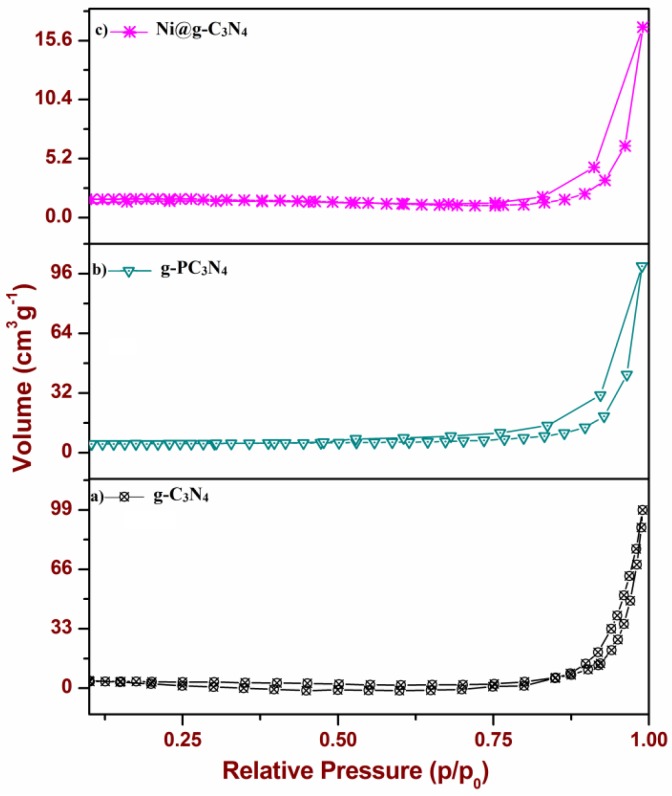
N_2_ adsorption-desorption isotherm and pore size distribution of: (**a**) g-C_3_N_4_; (**b**) g-PC_3_N_4_; (**c**) 5%Ni@g-PC_3_N_4_.

To determine the surface properties of the synthesized materials, N_2_ adsorption-desorption isotherms were determined by using multilayer Brunauer–Emmett–Teller (BET) adsorption-desorption theory. As seen in Figure 5, the adsorption-desorption isotherms were of type IV, suggesting the mesoporous nature of the materials [31]. The BET surface area (*S*_BET_), total pore volume (*V*_p_) and mean pore diameter (*r*_p_) of g-C_3_N_4_ were found to be 14.67 m^2^·g^−1^, 0.15 cm^3^·g^−1^ and 4.21 nm, while for g-PC_3_N_4_ these values were determined to be 24.59 m^2^·g^−1^, 0.14 cm^3^·g^−1^ and 3.83 nm, respectively. For the 5%Ni@g-PC_3_N_4_ surface area (*S*_BET_), the total pore volume (*V*_p_) and mean pore diameter (*r*_p_) were 48.62 m^2^·g^−1^, 0.17 cm^3^·g^−1^ and 2.64 nm, respectively. The change in the surface properties clearly indicated the grafting of nickel nanoparticles between the sheets of carbon nitride (Figure 5). The higher surface area provides more sites for reaction to proceed on the surface of the Ni@g-PC_3_N_4_ catalyst.

**Figure 6:**
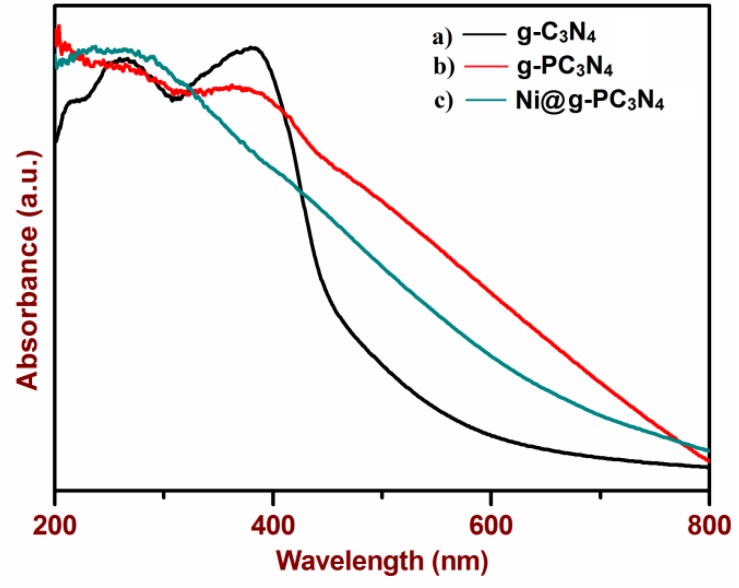
Ultraviolet--visible (UV-Vis) absorption spectra of: (**a**) g-C_3_N_4_; (**b**) g-PC_3_N_4_; (**c**) 5%Ni@g-PC_3_N_4_.

Electronic and optical properties are of fundamental importance to determine the photo activity of a catalyst. The absorption properties of synthesized materials were determined by UV-visible (UV-Vis) spectroscopy. The UV-visible spectrum of pure g-C_3_N_4_ shows an absorption spectrum similar to a typical semiconductor absorption spectrum between 200–450 nm, originating from the charge transfer from a populated valence band of nitrogen atom (2p orbitals) to a conduction band of carbon atom (2p orbitals) of carbon nitride [32]. An additional sharp peak at 256 nm, attributed to the aromatic ring’s π → π* transition, was observed. Another intense peak at 384 nm, due to the n → π* transitions caused by the electron transfer from a nitrogen nonbonding orbital to an aromatic anti-bonding orbital, was also present in the UV-Vis spectrum. The band tailing from 410 to 500 nm suggests a slight visible light absorption capacity of carbon nitride (Figure 6a). After doping with phosphorous atoms, the absorption band due to the nitrogen nonbonding to aromatic antibonding (n → π*) transition was diminished due to the replacement of carbon atoms with phosphorous, whereas the absorption pattern was found to be redshifted due to the better charge contribution by loosely bound electrons of phosphorous in the aromatic conjugated system (Figure 6b) [33]. After incorporation of nickel nanoparticles (5%Ni@g-PC_3_N_4_), the material exhibited lower absorbance in the visible region as the nickel nanoparticles have lower absorbance in the visible region, and therefore reduce the absorption profile in the visible region (Figure 6c).

**Figure 7:**
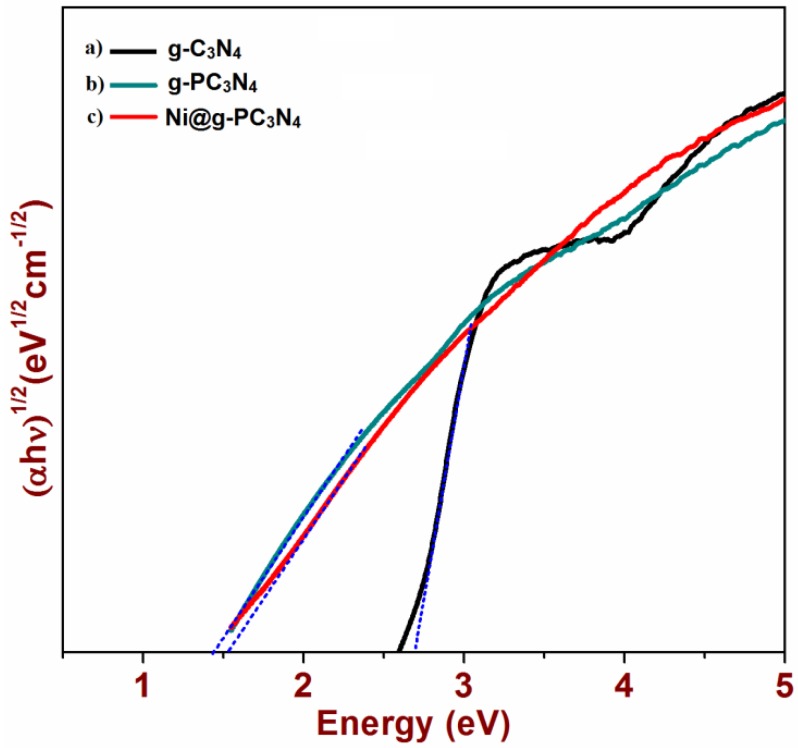
Tauc plots of (**a**) g-C_3_N_4_; (**b**) g-PC_3_N_4_; (**c**) Ni@g-PC_3_N_4_. α: absoption coefficient; hν: energy of incident photon.

In order to confirm the visible light absorption of the synthesized photocatalysts, a Tauc plot was obtained as shown in Figure 7. From the Tauc plot, the band gap value for the g-C_3_N_4_ was determined to be 2.69 eV due to the uneven synthesis of sheets, and was well in concordance with the existing literature [18]. After doping with phosphorous atoms (g-PC_3_N_4_), the value of the band gap decreased to 1.45 eV, which may be due to the displacement of carbon atoms by the phosphorous atoms in the triazine ring skeletons of carbon nitride. For the 5%Ni@g-PC_3_N_4_, a band gap value of 1.52 eV was calculated, which is nearly similar to that of g-PC_3_N_4_.

**Figure 8:**
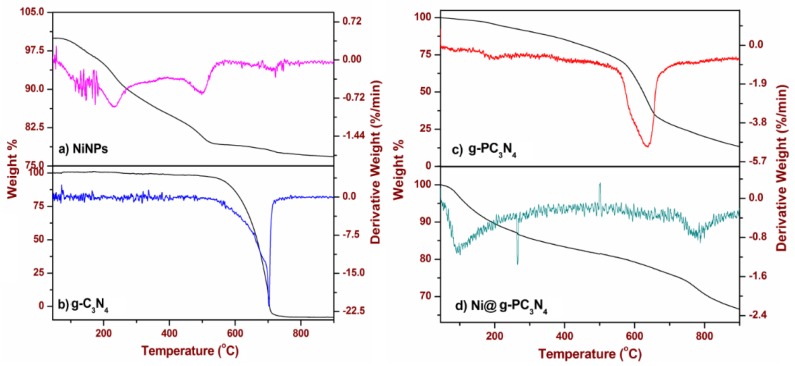
Thermogravimetric analysis (TGA) spectra of: (**a**) NiNPs; (**b**) g-C_3_N_4_; (**c**) g-PC_3_N_4_; (**d**) 5%Ni@g-PC_3_N_4_.

The thermal stability of the synthesized materials was examined by thermogravimetric analysis (Figure 8). A thermogravimetric analysis (TGA) pattern of pristine nickel nanoparticles showed steady weight loss (approx. 15%) from 100 to 500 °C, which may be due to loss of the residual organic template (CTAB) and loss of other oxygen-carrying functionalities during the crystallization step (Figure 8a). The thermogram of g-C_3_N_4_ exhibited a sharp weight loss in the range of 560 to 720 °C (Figure 8b). The P-doped g-C_3_N_4_ displayed a similar weight loss pattern as g-C_3_N_4_ (Figure 8c). For the 5%Ni@g-PC_3_N_4_, a small weight loss at around 100 °C was observed due to the loss of moisture, followed by steady weight loss up to 700 °C due to the loss of template in nickel and the slow degradation of the phosphorous-doped sheets. At 770 °C, a sharp weight loss was observed due to the complete degradation of P-doped carbon nitride sheets (Figure 8d).

### 2.2. Photocatalytic Reduction Reaction

The photocatalytic activity of the synthesized NiNPs, g-C_3_N_4_, g-PC_3_N_4_ and 2–7.5%Ni@g-PC_3_N_4_ catalysts was tested for the reduction of nitrobenzene as a model substrate using hydrazine monohydrate as a proton source under visible light irradiation. The results of these optimization experiments are summarized in Table 1. There was no conversion obtained with pristine nickel nanoparticles (NiNPs) (Table 1, entry 1). However, the yield of product was found to be 24.6 % when g-C_3_N_4_ was used as a photocatalyst (Table 1, entry 2). In case of P-doped g-PC_3_N_4_, the yield of aniline increased up to 54.2%, which clearly indicated the promoting effect of P-doping due to the better visible light absorption (Table 1, entry 3). Furthermore, the yield of aniline increased to manifold by using Ni@g-PC_3_N_4_ and, after 8 h of visible light irradiation, the yield reached 96.5% (Table 1, entries 4–6). This increase in the reaction rate and product yield can be explained because the nickel nanoparticles work as electron sinks, and the photogenerated electrons flow from conduction band of g-PC_3_N_4_ to nickel nanoparticles. This makes the electron unavailable for the recombination and therefore increases the activity of the catalyst. We also determined the optimum nickel content in the photocatalyst. It was found that among all the synthesized Ni@g-PC_3_N_4_ nanocomposites having variable nickel contents (2–7.5 wt % Ni), the nanocomposite having 5 wt % nickel nanoparticles was the most active and afforded maximum product yield (Table 1, entry 5). In the case of 2%Ni, the yield of product was lower, whereas no significant enhancement was observed with increasing the concentration of Ni beyond 5% (Table 1, entry 6). Furthermore, the reaction did not take place in dark conditions at ambient temperature by using g-C_3_N_4_, g-PC_3_N_4_ and Ni@g-PC_3_N_4_ photocatalysts, which clearly revealed that the reaction was visible light promoted (Table 1). The use of hydrazine hydrate was found to be essential as it provided required protons and no product was formed in the absence of hydrazine hydrate (Table 1, entry 5).

Based on these optimization experiments, 5%Ni@g-PC_3_N_4_ was selected as the optimum catalyst for further studies. The reaction was further generalized for various substituted nitrobenzenes and the results are summarized in Table 2. It can be seen that the substituent effect did not play much of a role and excellent product yields were obtained in all cases within 8 to 10.5 h.

**Figure 9:**
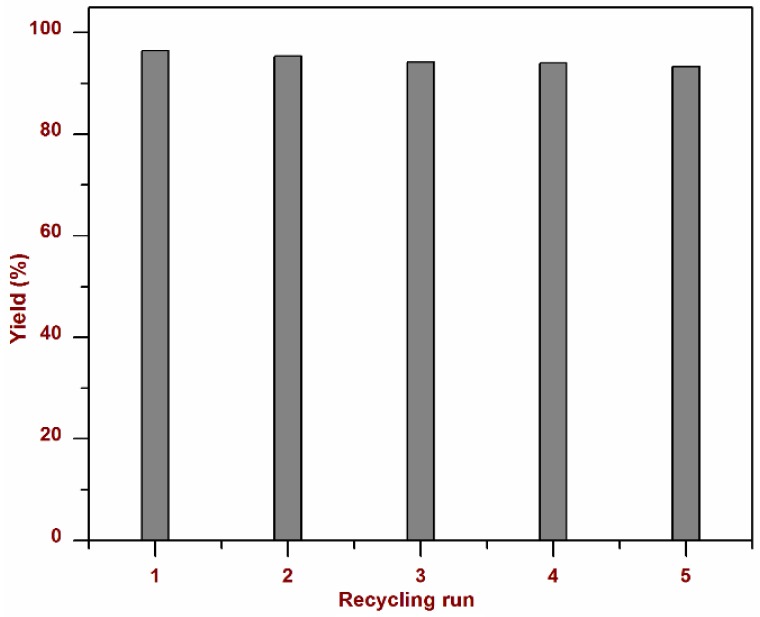
Results of recycling experiments.

The experiments were performed to probe the heterogeneous nature and reusability of the photocatalyst. After the completion of the reaction, the photocatalyst was recovered by centrifugation, washed with methanol and dried at 50 °C. The recovered photocatalyst was reused for five subsequent runs under described experimental conditions. No significant loss was observed in the activity of the recycled catalyst, and the product yield remained almost unchanged even after five recycling experiments, which confirmed the true heterogeneous nature of the developed photocatalyst. Furthermore, an inductively coupled plasma-atomic emission spectrometry (ICP-AES) analysis of the photocatalyst after five recycling experiments showed a nickel content of 4.92 wt %, which is nearly similar to that of a fresh catalyst (4.98 wt %). These results confirmed that the developed photocatalyst was truly heterogeneous in nature and had not shown any detectable leaching during the reaction (Figure 9).

**Scheme 3:**
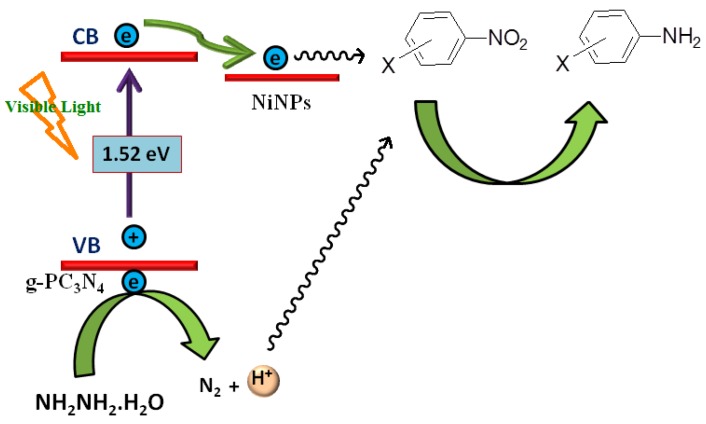
Plausible mechanism on the basis of the band gap structure for the visible light reduction of nitrobenzenes by Ni@g-PC_3_N_4_ photocatalyst. CB: conduction band; VB: valence band.

Although the exact mechanism of the reaction is not clear at this stage, we assume that the generation of electron-hole pairs after the absorption of visible light is the first step to initiate the reaction [34]. Since the optical band gap of P-doped g-PC_3_N_4_ is 1.52 eV, it can generate electrons and holes in its conduction and valence band, respectively. Electrons from the conduction band of P-doped carbon nitride get transferred to nickel nanoparticles due to lower Fermi level, which therefore work as electron sinks and capture the photogenerated electrons and prevent back charge recombination [35]. The electrons from NiNPs are transferred to nitrobenzene, which initiate a one electron reduction step [36]. The holes in the valence band of g-PC_3_N_4_ oxidize hydrazine hydrate and generate electrons and protons along with nitrogen gas. The protons were used for the hydrogenation of activated molecules of nitrobenzene. As for the reduction of nitrobenezene to aniline, six protons and six electrons are required, so 3/2 NH_2_NH_2_·H_2_O mole were consumed per mole of reactant (Scheme 3).

## 3. Experimental Section

### 3.1. Materials

Dicyandiamide (99%), 1-butyl-3-methylimidazolium hexafluorophosphate [BmimPF_6_] (≥98.5%), nickel chloride hexahydrate (≥98%), cetyltrimethylammonium bromide (≥98%) and sodium borohydride (≥98.0%) were purchased from Aldrich (St. Louis, MO, USA) and used as received. All substrates and solvents were purchased from Merck India Ltd (Mumbai, Maharashtra, India) and used without further purification.

### 3.2. Characterizations

The rough morphological surface properties of materials were determined with the help of scanning electron microscopy (SEM) using FE-SEM (Jeol Model JSM-6340F (Tokyo, Japan). For elaborating the fine structure of the catalysts, high-resolution transmission electron microscopy (HRTEM) was used and HRTEM images were collected on FEI-TecnaiG^2^ Twin TEM (Hillsboro, OR, USA) operating at an acceleration voltage of 200 kV. For sample preparation, a very dilute suspension of material was deposited on a carbon coated TEM grid. TEM images were processed on GATAN micrograph software (Munchen, Germany). The vibration spectra (FT-IR) of samples were recorded on a Perkin–Elmer spectrum RX-1 IR spectrophotometer (Waltham, MA, USA) using a potassium bromide window. X-ray diffraction patterns for determining the phase structure and crystalline properties of the materials were obtained on a Bruker D8 Advance diffractometer (Billerica, MA, USA) working at 40 kV and 40 mA with Cu K_α_ radiation (λ = 0.15418 nm). Nitrogen adsorption-desorption isotherms for calculating surface properties like Brunauer-Emmet-Teller surface area (*S*_BET_), Barret-Joiner-Halenda (BJH) porosity, pore volume, *etc.,* were acquired on a *V*_P_. Micromeritics ASAP2010 (Norcross, GA, USA) at 77 K. UV-Vis absorption spectra of solid samples were collected on a Perkin Elmer lambda-19 UV-VIS-NIR spectrophotometer (Waltham, MA, USA) by making 1 mm thick pellets using BaSO_4_ as reference. To check the thermal stability of materials, thermogravimetric analysis (TGA) was performed by using a TA-SDT Q-600 thermal analyser (Champaign, IL, USA) in the temperature range of 45 to 800 °C under nitrogen flow with a heating rate of 10 °C/min. To determine the nickel content in the synthesized composites, ICP-AES analysis was performed by using inductively coupled plasma atomic emission spectrometer (ICP-AES, DRE, PS-3000UV, Leeman Labs Inc., Hudson, NH, USA). Samples for ICP-AES were made by digesting a calculated amount of samples with nitric acid followed by filtration and making volume up to 10 mL by adding deionized water.

### 3.3. Synthesis of Nickel Nanoparticles [26]

Nickel nanoparticles were synthesized by following a literature procedure. In brief, to an aqueous suspension of nickel chloride hexahydrate (0.152 mmol, 0.019 g) and cetyltrimethyl ammonium bromide, CTAB (0.288 mmol, 0.105 g), an aqueous solution (1.5 mL) of NaBH_4_ (0.020 g, 0.526 mmol) was added dropwise. The mixture was vigorously stirred to obtain a black suspension. The particles were separated by centrifugation and washed with water several times.

### 3.4. Synthesis of Phosphorous-Doped Graphitic Carbon Nitride (g-PC_3_N_4_) [19,27]

Phosphorous-doped graphitic carbon nitride was synthesized by using BmimPF_4_ as a source of phosphorous. The phosphorous containing ionic liquid, BmimPF_4_ (0.5 g) was dissolved in water (6 mL) and stirred for 5 min. After that, dicyandiamide (1 g) was added to this solution and the mixture was heated at 100 °C until all the water had completely evaporated, which resulted in the formation of a white solid. The obtained solid was subjected to heating at a programmed temperature. Firstly the sample was heated up to 350 °C within 2 h, then the temperature was maintained at a constant for the next 4 h. The temperature was then raised to 550 °C in 1 h and then this temperature was maintained at a constant for the next 4 h. The sample was collected at room temperature.

### 3.5. Synthesis of Nickel Nanoparticles Decorated on Phosphorous-Doped Graphitic Carbon Nitride (Ni@g-PC_3_N_4_)

For the synthesis of nickel nanoparticle-decorated carbon nitride, nickel particles were added along with BmimPF_4_ and dicyandiamide during the synthesis step. Then the sample was heated at a programmed temperature as in the synthesis of P-doped carbon nitride.

### 3.6. Photocatalytic Reduction Experiment

To check the activity of the catalyst for the visible light mediated reduction, a 20 watt LED (Model No. HP-FL-20W-F-Hope LED Opto-Electric Co., Ltd (Shenzhen, China), λ > 400 nm) was used as a source of visible light. A round bottom flask was charged with 25 mg of 5%Ni@g-PC_3_N_4_ catalyst, 0.1 mmol of nitrobenzene and 1.0 mmol of hydrazine monohydrate in 10 mL solvent mixture of acetonitrile/DCM/methanol. After sonication for 10 min, the resulting mixture was irradiated under visible light. To monitor the progress of the reaction, the sample was withdrawn at a certain period of time and analyzed by gas chromatography-flame ionization detector (GC-FID). After the completion of the reaction, the solvent was removed by rotary evaporation and the product was isolated using column chromatography. The identification of product was done by gas chromatography-mass spectrometry (GC-MS) and ^1^H-nuclear magnetic resonance (^1^H-NMR).

## 4. Conclusions

We have synthesized a novel, highly efficient and visible light-active nickel nanoparticles-decorated P-doped carbon nitride for the selective hydrogenation of nitro compounds to the corresponding amines in the presence of hydrazine monohydrate as a proton donor. Due to P-doping, the developed photocatalyst absorbs the maximum part of the visible region and nickel nanoparticles work as an electron trap. The developed photocatalyst was found to be highly effective and afforded excellent product yields within 8–10.5 h at ambient temperature under visible light irradiation. Due to the heterogeneous nature of photocatalyst, it can be easily recovered and reused for further reactions without any significant decrease in its activity. The enhanced performance of the catalyst in the visible region can be explained on the basis of reduced band gap, which generates electron-hole pairs through the absorption of visible light. Photogenerated electrons are efficiently captured by nickel nanoparticles on the sheets and subsequently transferred to substrate molecule, while holes are used to oxidize hydrazine hydrate and extract required protons and electrons for the reaction.

## Figures and Tables

**Table 1:** Results of optimization experiments ^a^. NiNPs: nickel nanoparticles; g-C_3_N_4_: graphitic carbon nitride; g-PC_3_N_4_: phosphorous doped g-C_3_N_4_; Ni@g-PC_3_N_4_: nickel nanoparticles grafted on P-doped g-C_3_N_4_; TOF: turn over frequency.

Entry	Catalyst	Conditions	Time (h)	AnilineYield (%) ^b^	TOF (h^−1^)
1	NiNPs	Dark	24	-	-
		Visible light	-	-	-
2	g-C_3_N_4_	Dark	24	-	-
		Visible light	12	24.6	2.0
3	g-P-C_3_N_4_	Dark	24	-	-
		Visible light	12	54.2	4.5
4	2%Ni@g-PC_3_N_4_	Dark	24	-	-
		Visible light	8	82.0	10.2
5	5%Ni@g-PC_3_N_4_	Dark	24	Trace	-
		Visible light	8	96.5	12.1
		Visible light	24 ^c^	- ^c^	- ^c^
6	7.5%Ni@g-PC_3_N_4_	Dark	24	10	0.4
		Visible light	8	97.0	12.1

^a^ Reaction conditions: nitrobenzene, 0.1 mmol; catalyst, 25 mg; hydrazine monohydrate, 1 mmol; Irradiation, White cold 20 W light emitting diode (LED) λ > 400 nm, Power at reaction vessel 70 W/m^2^; ^b^ Isolated yield; ^c^ without hydrazine monohydrate.

**Table 2:** 5%Ni@g-PC_3_N_4_ catalyzed photoreduction of nitrobenzenes ^a^.

Entry	Reactant	Product	Time/h	Conversion (%) ^b^	Yield (%) ^c^	TOF (h^−1^)
1	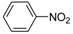	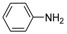	8.0	98.0	96.5	12.1
2	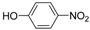	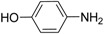	8.0	95.5	94.2	11.7
3	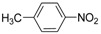	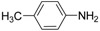	8.0	96.4	95.4	11.9
4	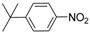	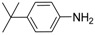	8.0	94.8	93.0	11.6
5	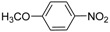	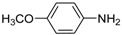	8.0	95.6	94.2	11.7
6	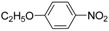	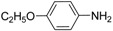	8.0	96.0	94.6	11.8
7	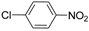	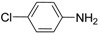	8.5	90.5	89.4	10.5
8	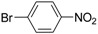	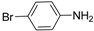	8.5	92.4	91.0	10.7
9	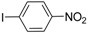	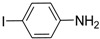	10.5	93.6	92.8	8.8
10	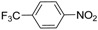	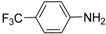	10.5	90.4	89.6	8.5

^a^ Reaction conditions: nitro compound, 0.1 mmol; catalyst, 25 mg; hydrazine monohydrate, 1 mmol; Irradiation, White cold 20 W LED λ > 400 nm, Power at reaction vessel 70 W/m^2^; ^b^ Conversion (Conv.) was determined with gas chromatography (GC); ^c^ Isolated yield.
